# Membranous nephropathy in a patient with ankylosing spondylitis: A case report

**DOI:** 10.1097/MD.0000000000008201

**Published:** 2017-10-27

**Authors:** Ruiying Chen, Fang Li, Qionghong Xie, Jun Xue, Lingyun Lai, Shaojun Liu, Liyin Zhang, Chuanming Hao

**Affiliations:** a Division of Nephrology, Huashan Hospital; b Department of Pathology, Shanghai Medical College, Fudan University, Shanghai, China.

**Keywords:** adalimumab, ankylosing spondylitis, membranous nephropathy

## Abstract

**Rationale:**

Renal complications in ankylosing spondylitis (AS) were rarely observed, and proteinuria associated with AS can be seen often due to amyloidosis in this kind of complications, while membranous nephropathy (MN) is seldom considered. This article reports a case of coexistence of AS and MN, to provide the exact relationship of these 2 entities and recognized some causes of renal involvement in AS.

**Patient concerns:**

A 44-year-old female presented with pain of the left leg for 4 years and pedal edema for 2 weeks.

**Diagnoses:**

AS was diagnosed according to the patient's clinical manifestation and sacroiliitis observed on computed tomography (CT) scan. Nephrotic syndrome was found and MN was diagnosed according to kidney biopsy in which thickened capillary loops were observed with light microscopy, granular deposits of IgG along the capillary wall were observed using immunofluorescence staining, and subepithelial electron-dense deposits were observed with electron microscopy. No other secondary causes of MN were found on extensive investigations.

**Intervention:**

Given the diagnoses, the patient received nonimmunosuppressive therapy for MN and adalimumab for AS.

**Outcomes:**

The patient got pain relief, as well as urinary protein reduction.

**Lessons:**

This case suggested a secondary MN in association with AS and the relationship between these 2 diseases needed more concern and further illumination.

## 1 Introduction

Ankylosing spondylitis (AS), a form of spondyloarthritis, is a chronic inflammatory disease of the axial skeleton manifested accompanied with back pain and progressive stiffness of the spine; this pain can also occur in the hips, shoulders, and peripheral joint. Extra-articular manifestations, including uveitis, may also happen. Renal involvement is uncommon in AS, only occurring in 5% to 13% of AS patients.^[1–3]^ The most commonly reported renal complications were secondary amyloidosis, followed by tubulointerstitial nephropathy and IgA nephropathy.^[4]^ Membranous nephropathy (MN) in the patients with AS is extremely rare. In a cohort of 93 patients with AS and renal involvement, only 1 was MN.^[2]^ So far, there are only a few cases showing MN associated with AS, and none of them straightly connected MN to AS.^[5–9]^ Here, we reported a case that may show tighter connection between these 2 diseases, recognizing the causes of renal involvement in AS.

## 2 Case report

A 46-year-old female presented with swelling of the feet for 2 weeks. Her laboratory examination revealed proteinuria (+++) without hematuria. The patient had suffered from pain of the left leg, most often at night since 2012, accompanied by morning stiffness of which alleviated after activities. She had been using nonsteroidal anti-inflammatory drugs (NSAIDs) for symptomatic pain relief. Her urine test was normal 2 years ago, and the retroperitoneal nodes showed mild enlargement. She had a family history of mildly enlarged spleen of her mother.

Physical examination showed enlarged inguinal lymph nodes and mild pedal edema. Laboratory results revealed mild microcytic hypochromic anemia (hemoglobin 99 g/L) with normal ferritin but decreased transferrin saturation. The album and globulin were 20 and 44 g/L, respectively. All types of globulin were elevated. The serum creatinine was 39 μmol/L, and cholesterol was 7.46 mmol/L. The urine test showed hematuria (+) and proteinuria (+++). A 24-h urine examination revealed protein excretion of 7.73 g. Serum phospholipase A2 receptor (PLA_2_R) antibodies were negative.

**Figure 1:**
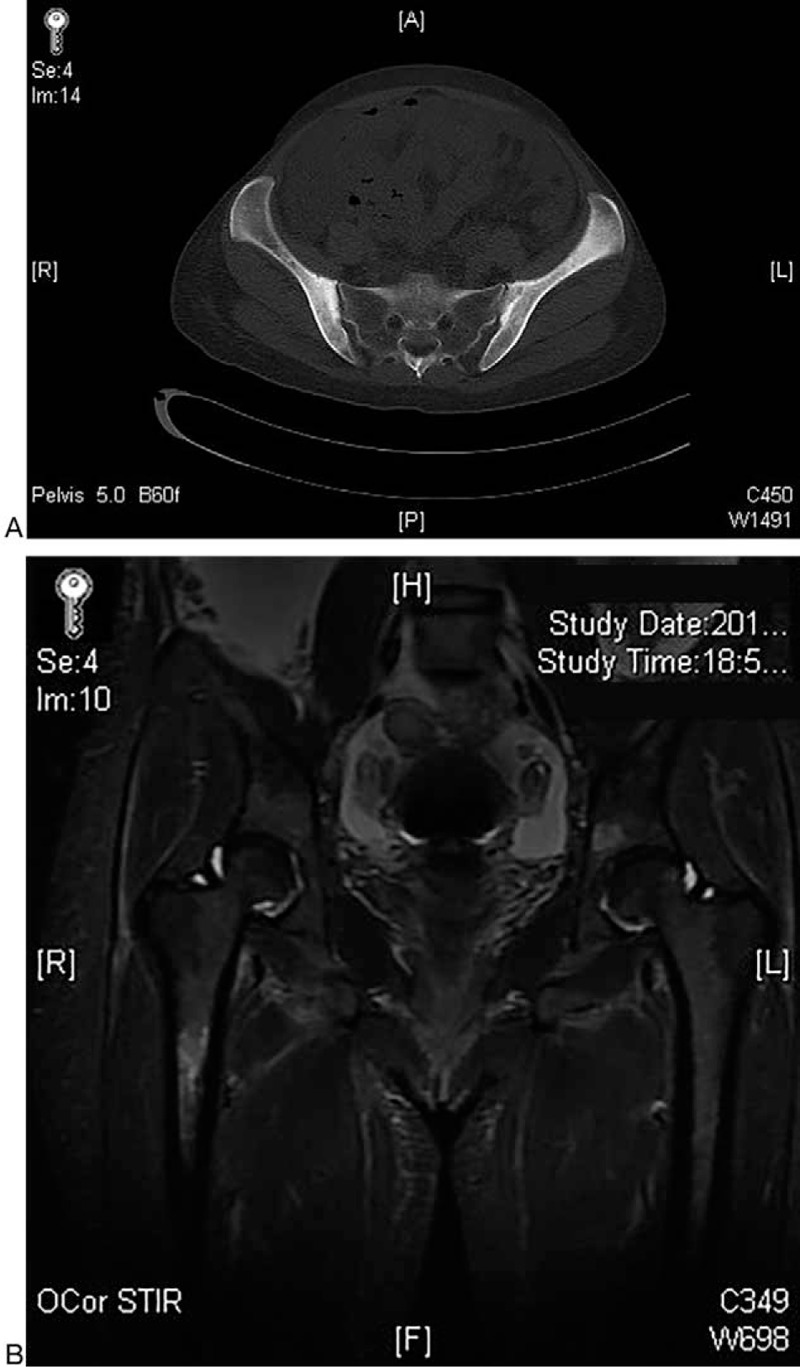
Imaging studies. (A) The ilia show mildly expansile change with thickened and blurred cortex on contrast-enhanced computed tomography (CT) scan, which indicates ossification and bilateral sacroiliitis, greater on the left. (B) Edema of bone marrow was observed in the right proximal femur, which manifested as increased signal intensity in the short tau inversion recovery (STIR) images.

The immunologic tests were all negative and cancer markers showed no clinically significant increase. Although the levels of C-reactive protein (CRP) and erythrocyte sedimentation rate (ESR) were significantly increased that indicated inflammation, there was no evidence of chronic infectious diseases, including active tuberculosis, except of strongly positive for T-SPOT.TB assay. Radiology evaluation revealed bone marrow edema in right proximal femur and sacroiliitis (Fig. 1), whereas the bone scan showed high radiation uptake, suggesting inflammation status. Chest contrast-enhanced computed tomography (CT) scan showed atelectasis of the left lower lobe and pleural effusion, with no signs of pneumonia. CT and sonography both showed ascites and enlarged spleen (133∗51 mm), supraclavicular, and inguinal lymph nodes (maximal 15∗5 mm).

**Figure 2:**
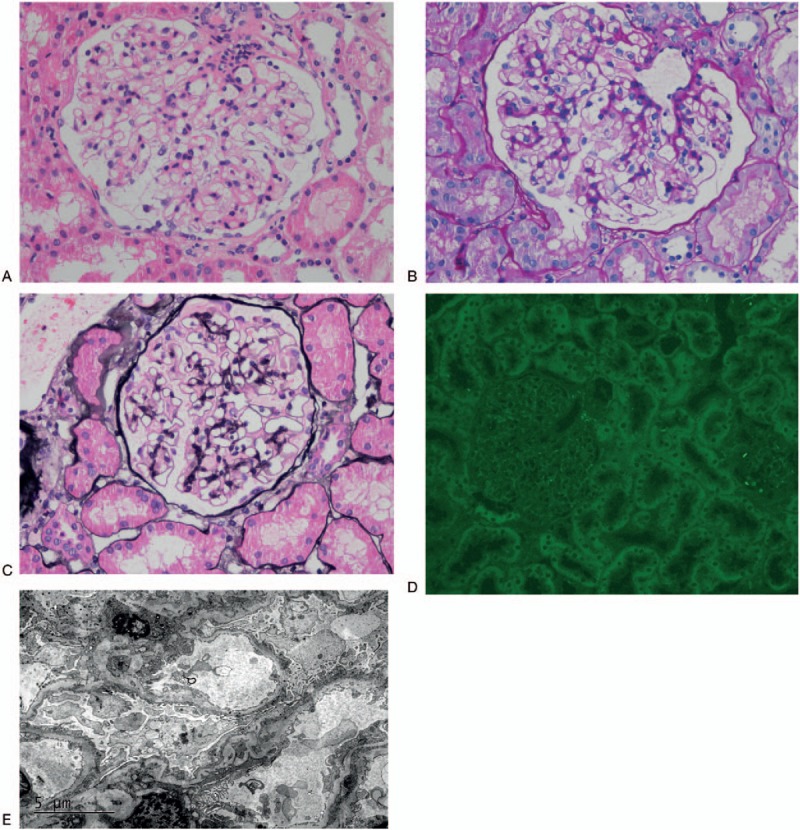
Renal biopsy. Histological analysis highlighted the thickening and stiffness of the capillary basement membrane (A, Hematoxylin and eosin staining, X400; B, Periodic acid Schiff, X400). PASM staining showed vacuoles in the thick capillary wall without significant spikes (C, X400). PLA_2_R staining was negative (D, X400). Electron microscopy showed subepithelial granular electron-dense deposits, with some even penetrating the basement membrane. Overlying podocyte foot processes effaced obviously (E).

When nephrotic syndrome was diagnosed, the patient subsequently underwent percutaneous renal biopsy. Light microscopy showed 18 glomeruli with thick-appearing capillary loops without proliferative activity or crescents (Fig. 2A, B). Trichrome staining results suggested the presence of red subepithelial deposits. Periodic acid-silver metheramine (PASM staining) demonstrated thickening capillary loops with vacuoles in some sections, although subepithelial spikes were not seen (Fig. 2C). There was no evidence of interstitial fibrosis, tubular necrosis, or atrophy. Arteries showed mild intimal thickening. By immunofluorescence staining, subepithelial deposits were visualized as fine granular positivity along the capillary wall, which were strongly positive staining of IgG(+++), with slightly positive staining for κ and λ but negative staining for IgA, IgM, C3, and C1q. Renal PLA_2_R staining was negative (Fig. 2D). Ultrastructural evaluation using electronic microscope showed subepithelial electron-dense deposits, with some even throughout the basement membrane. There were no obvious changes in the mesangium or subendothelial area. The overlying podocyte foot processes were diffusely effaced (Fig. 2E). All the biopsy findings confirmed the diagnosis of MN (stage|+) and suggested the possibility of a secondary etiology. For MN, nonimmunosuppressive therapy was undertaken. According to the typical clinical signs of arthralgia and radiologic findings of sacroiliitis, the diagnosis of AS was also considered and celecoxib was still needed for pain relief. In addition, inguinal lymph node biopsy and bone marrow biopsy were undertaken to exclude lymphoma and other hematological malignances. The final diagnoses were AS and MN (probably secondary to AS). The patient was then recommended to Rheumatism Department for treatment of AS.

**Figure 3:**
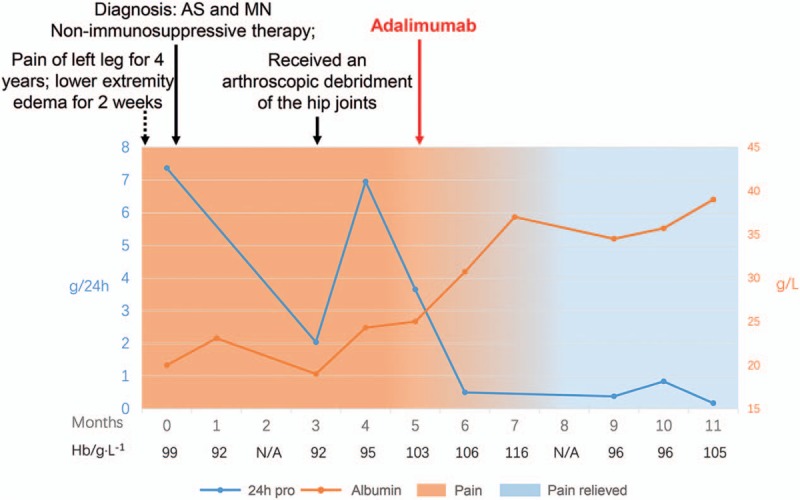
Timeline.

Three months later, the patient had no remission of nephrotic syndrome with continuous using of celecoxib. She did not receive any other medicine against AS. To stop taking NSAIDs, she received an arthroscopic debridment of the hip joints, but got no pain relief. Then, tumor necrosis factor (TNF)-alpha antagonist Adalimumab (40 mg/2 weeks) was used. Since then, the pain disappeared completely and no any other medication was undertaken for pain relief. The 24-h urine protein decreased rapidly to a partial remission level, while album increased to a normal level during the following 3 months. Anemia was also relieved, with the falling level of CRP and ESR. Timeline of diagnostic and therapeutic procedures is summarized in Fig. 3.

## 3 Discussion

MN is the most common cause of nephrotic syndrome in nondiabetic adults, which was characterized by glomerular basement membrane thickening without significant hypercellularity, and the presence of subepithelial electron-dense deposits. Although MN is most often idiopathic, it has been documented to be secondary to infectious diseases, autoimmune diseases, malignancies, and the use of certain drugs such as penicillamine, gold, captopril, and NSAIDs.^[10]^ MN associated with AS is believed to be uncommon. There were only 5 cases by searching on PubMed website, 2 of which the patients had concurrent rheumatoid arthritis and were exposed to gold salt.^[5–9]^ Therefore, it is difficult to get conclusion that MN could be secondary to AS. In our case, the causal relationship between these 2 diseases appeared highly possible because therapy for AS has been shown to be effective in improving both AS and nephrotic syndrome simultaneously. Furthermore, renal PLA_2_R, as well as serum PLA_2_R antibodies, which is supposed to be a marker of primary MN, was negative.

MN secondary to some primary diseases is hard to diagnosed because of the following reasons. First, these primary diseases are low in prevalence or are hard to be diagnosed. Second, although the primary diseases are common, MN secondary to these diseases is unusual, such as syphilis or tuberculosis-associated MN.^[11–14]^ Third, there is no effective diagnostic method for these secondary MNs, such as hepatitis B virus-associated MN.^[15]^ Although AS is a common disease, the appearance of MN in AS patients is very rare, and resulted in the lack of epidemiologic evidence supporting AS-associated MN. In a study including 1034 AS patients, only 1 patient suffered from MN, significantly less than amyloidosis (n = 58) and IgA (n = 28).^[2]^ In addition, no ideal method is available for diagnosing AS-associated MN. Whether MN is secondary to AS or just a concurrent disease remains undetermined. In the 5 cases reported by others, no direct evidence linked MN to AS, as 2 patients were accompanied by rheumatoid arthritis and taking gold salt, and 4 patients were taking NSAIDs for pain relief.^[5–9]^ In our case, the patient was diagnosed as AS according to the history of chronic night pain in the lower back and hips and the typical radiological manifestation of sacroiliitis. The MN appeared in the active stage of AS manifesting as unrelieved pain without NSAIDs and inflammatory anemia. It was then alleviated after AS remission, treated by TNF-alpha antagonist that revolutionized the management of AS but did not show proven efficacy in reducing proteinuria in idiopathic MN.^[16–20]^ Both the negative PLA_2_R and the finding that electron-dense deposit run through the whole basement membrane by electron microscope also suggested secondary MN. All these clinical findings clearly suggested that MN was secondary to AS in this case.

Before diagnosing AS-associated MN, several diseases suspected that linking to MN should be excluded. First is the NSAIDs. The present patient has taken NSAIDs for 2 years, which was widely accepted as a cause of secondary MN. However, NSAIDs-associated MN was not plausible, as nephrotic syndrome was not relieved after NSAIDs withdrawal or replacement with celecoxib.^[21,22]^ More importantly, the renal pathological result did not support NSAIDs-associated MN. Interestingly, PLA_2_R-positive staining has been reported in 4 of 5 cases with NSAIDs-associated MN,^[10]^ while renal and serum PLA_2_R antibodies were both negative in our case. Second is lupus. Lupus is sometimes associated with MN in woman. However, it was less likely here because neither the clinical manifestation fulfilled the criteria for system lupus erythematous nor the renal pathology showed “full-house” immunofluorescence pattern. Although anemia was found in this patient, it was probably associated with chronic inflammation caused by AS and improved after AS remission. Last but not the least is tuberculosis. MN caused by tuberculosis should also be differentiated here, as the T-SPOT test of the patient showed strongly positive.^[12–14]^ It is important to consider the possibility because of the potentiality to worsen underlying tuberculosis by the immunosuppressive therapy for MN. In this patient, there were no active signs of tuberculosis, including symptoms and imaging test. Her MN finally alleviated in the absence of management of tuberculosis, which supported nontuberculosis-associated MN.

In summary, now there was no study, as well as epidemiologic evidence, that directly demonstrated the causal relationship between MN and AS. AS is an under-recognized secondary cause of MN. In this case, we presented a patient showing strong correlation between these 2 diseases. MN may occur in the active stage of AS and the treatment of AS by TNF-alpha antagonist was useful for MN remission.
